# Genetic Diversity of *Bartonella henselae* in Human Infection Detected with Multispacer Typing

**DOI:** 10.3201/eid1308.070085

**Published:** 2007-08

**Authors:** Wenjun Li, Didier Raoult, Pierre-Edouard Fournier

**Affiliations:** * Université de la Méditerraneé, Marseille, France

**Keywords:** Bartonella, genetic heterogeneity, gene sequences, multispacer typing, genotyping, research

## Abstract

We applied multispacer typing (MST) by incorporating 9 variable intergenic spacers to *Bartonella henselae* DNA detected in lymph node biopsy specimens from 70 patients with cat-scratch disease (CSD), in cardiac valve specimens from 2 patients with endocarditis, and in 3 human isolates from patients with bacillary angiomatosis, CSD, and endocarditis. Sixteen MST genotypes were found, 5 previously identified in cats and 11 new. Of the studied DNA, 78.7% belonged to 2 genotypes, which were phylogenetically organized into 4 lineages. Human strains were mostly grouped within 2 lineages, previously identified as Marseille and Houston-1. Our results suggest a greater genetic diversity in human-infecting *B. henselae* than what has previously been evaluated by using other genotyping methods. However, the diversity is not significantly different from that of cat strains. MST is thus a suitable genotyping tool for evaluating the genetic heterogeneity of *B. henselae* among isolates obtained from human patients.

## Abstract 2

This techniqueis a suitable tool for evaluating the genetic diversity of *B. henselae* human isolates.

*Bartonella henselae*, first identified in 1990 and characterized as a new species in 1992, is a gram-negative, fastidious bacterium associated with cats. *B. henselae* infection in cats is usually asymptomatic, but infected cats may remain bacteremic for long periods, thus playing a major role as a reservoir for the bacterium (*1*,*2*). Transmission of *B. henselae* among cats may be mediated by the cat flea, *Ctenocephalides felis* (*3*), and to humans by cat scratches or bites (*4*). *B. henselae* infection in humans exhibits a variety of clinical syndromes including the most common, cat-scratch disease (CSD) (*5*), endocarditis (*6*), bacillary angiomatosis (*7*) and peliosis hepatis in immunocompromised patients (*8*), and other less frequent manifestations (*9*). *B. henselae* has also been detected in various domestic or wild animals, including dogs, lions, panthers, and cheetahs (*10*). More recently, *B. henselae* was detected in the porpoise, a marine mammal (*11*).

Because *B. henselae* has a complex and expanding host or reservoir system and has been associated with a rapidly increasing spectrum of clinical syndromes (*12*), epidemiologic survey and exploration of population structure of this organism are critical. The following techniques have been used for this purpose: pulsed-field gel electrophoresis (PFGE); restriction fragment length polymorphism; enterobacterial repetitive intergenic consensus (ERIC)–PCR; repetitive extragenic palindromic (REP)–PCR; infrequent restriction site (IRS)–PCR (*10*,*13*–*18*); DNA sequencing–based approaches represented by sequences of 16S rDNA (*19*,*20*), *ftsZ* (*21*), *gltA* (*22*), 35-kDa protein–encoding (*23*), *groEL,* and *pap*31 genes (*24*); and the 16S-23S intergenic spacer (*25*). These investigations gradually offered insight into the population structure of *B. henselae* and allowed several genetic groups to be identified. Initially, *B. henselae* isolates were classified within 2 16S rRNA–based genotypes, I and II, and 2 serotypes, Marseille and Houston-1 (*20*,*23*,*26*). Studies that used *gltA*, *groEL,* and *pap*31 gene sequence analysis, arbitrarily primed–PCR, ERIC-PCR, IRS-PCR, PFGE, or multilocus sequence typing (MLST) were congruent with serotypes, but not with genotype I and II classification (*13*,*22*–*24*,*27*–*29*). Altogether, *B. henselae* isolates were found to be distributed within 3 distinct lineages (Marseille, Houston-1, and Berlin-2), and the 16S rRNA gene was not a sensitive marker of the clonal divisions of *B. henselae*. This suggested that a 16S rRNA recombination occurred in this species that may be facilitated by the coexistence of several strains in the blood of cats (*27*).

Several studies have suggested that human-associated isolates were less genetically heterogeneous than cat isolates (*22*,*27*,*30*), and a small group of cat isolates were hypothesized to be the source of human infection (*22*,*27*). However, the limited discriminatory power of the available genotyping methods, with a maximum of only 7 genotypes identified (*27*), and the small number of human isolates studied prevented the population structure and the genetic relationship between cat and human isolates of *B. henselae* to be reliably investigated. Recently, we applied multispacer typing (MST), using 9 highly variable intergenic spacers, to 126 *B. henselae* cat isolates and identified 39 MST genotypes, which made it the highest resolution typing system (available from http://ifr48.timone.univ-mrs.fr/mst_bhenselae/mst) (*31*). In this study, we applied MST, based on sequences from the 9 previously described variable intergenic spacers (*31*), to 75 human-infecting *B. henselae* isolates to evaluate their genetic diversity and compare it to that of cat isolates.

## Materials and Methods

### Study Design

We included in the study, when available, lymph node biopsy specimens and cardiac valve specimens from patients with a clinical diagnosis of CSD or endocarditis who had been referred to our laboratory from 2004 to 2006. All specimens were then frozen at –80°C for further culture and molecular detection of *Bartonella* species. *B. henselae* was detected by using PCR targeting the 16S-23S rRNA spacer (ITS) and *pap*31 gene (*24*,*25*). In addition, 3 human *B. henselae* isolates—type strain Houston-1, isolated from the blood of a patient with bacillary angiomatosis in USA; type strain Marseille, isolated from the lymph node biopsy specimen of a patient with CSD in France; and strain URBHTOIE49, isolated from the valve biopsy specimen of a patient with endocarditis in France (*26*)—were incorporated in this study. All *B. henselae* isolates and PCR amplicons were tested by MST by using 9 variable intergenic spacers named S1-S9, from the most to the least variable among cat isolates (*31*). When available, the 16S rRNA genotypes of studied strains were indicated (Appendix Figure).

### Culture Conditions of *B. henselae* Human Isolates

*B. henselae* isolates were cultivated on blood agar (bioMérieux, Marcy l’Etoile, France) at 37°C in 5% CO_2_ (Genbag CO_2_ System, bioMérieux). After a 5-day incubation period, *Bartonella* cells were collected for DNA extraction.

### DNA Preparation

Total genomic DNA was extracted from the 3 studied *B. henselae* isolates by using the Chelex procedure as previously described (*32*). For patients’ specimens, we used the QIAamp Tissue kit (QIAGEN, Hilden, Germany) to extract the total genomic DNA, as described by the manufacturer.

### PCR Amplification and Sequencing

ITS and *pap*31 gene PCR amplifications were performed by using the previously described conditions and the 16SF-23S1 (*25*) and PAPn1-PAPn2 (*24*) primer pairs, respectively. For amplification and sequencing of the 9 intergenic spacers, we used the same primers as in our previous study (*31*), except for primers amplifying spacers S1 and S8, which caused unspecific amplifications from DNA extracted from human specimens. One new set of primers was designed to amplify each of these 2 spacers, as indicated in the  Appendix Table.

These new primers also amplified the whole intergenic spacer and thus did not affect genotype comparison between human and cat strains. All primers were obtained from Eurogentec (Seraing, Belgium). PCRs were performed in a PTC-200 automated thermal cycler (MJ Research, Waltham, MA, USA). One nanomolar concentration of each DNA preparation was amplified in a 25-μL reaction mixture containing 50 pmol/L of each primer; 200 μmol/L (each) dATP, dCTP, dGTP, and dTTP (Invitrogen, Gaithersburg, MD, USA); 1.5 U of HotstarTaq DNA polymerase (QIAGEN); 2.5 μL 10× PCR buffer; and 1 μL 25 mmol/L MgCl_2_. The following conditions were used for amplification: an initial 15-min step at 95°C was followed by 40 cycles of denaturation for 30 s at 94°C, annealing for 30 s at 55°C, and extension for 1 min at 72°C. Final amplification was completed by holding the reaction mixture for 5 min at 72°C to allow complete extension of the PCR products. PCR products were purified by using the MultiScreen PCR filter plate (Millipore, Saint-Quentin-en-Yvelines, France) as recommended by the manufacturer. Amplicons were sequenced in both directions by using the BigDye 1.1 chemistry (Applied Biosystems, Foster City, CA, USA) on an ABI 3130XL automated sequencer (Applied Biosystems) as described by the manufacturer. To avoid contamination, no positive control was used. Sterile water was used as a negative control in each PCR. Sequences from each DNA sample were checked twice in both directions to ensure the reliability of the MST method.

### Sequence Analysis

Nucleotide sequences were edited by using the Autoassembler package (PerkinElmer, Waltham, MA, USA). For each intergenic spacer, a genotype was defined as a sequence exhibiting unique mutations, which were checked by sequencing the corresponding spacers 3 times. MST genotypes were defined as unique combinations of the 9 spacer genotypes.

### Phylogenetic Analysis

For the phylogenetic analysis, we concatenated sequences from the 9 studied spacers. We included in the analysis the 39 MST genotypes previously obtained from the MST analysis of cat isolates (*31*) and those, when different, obtained in the present study. Multiple alignment of sequences was carried out by using CLUSTALW software (*33*). Phylogenetic relationships were obtained by using the neighbor-joining and maximum parsimony methods within the MEGA 2.1 software (*34*), and the maximum likelihood method within the BioEdit software (available from www.mbio.ncsu.edu/bioedit/bioedit.html).

### Statistical Analysis

The genotypic diversities of human and cat strains of *B. henselae* were compared by using the Fisher exact test within the Epi Info 6.0 software (Centers for Disease Control and Prevention, Atlanta, GA, USA). A difference was considered statistically significant when p<0.01.

## Results

### Genotypic Distribution of 75 *B. henselae* Strains Detected in Humans

From January 2004 to May 31, 2006, *B. henselae* was detected in 70 lymph node biopsy specimens from 70 patients with CSD and 2 cardiac valve specimens from 2 patients with endocarditis by using PCRs targeting the ITS and *pap*31 gene. Among the 72 *B. henselae*–positive samples, 71 were obtained from patients living in France, and the remaining specimen was from a cardiac valve biopsy from a patient living in Guadeloupe Island in the West Indies. When applying MST to the 72 *B. henselae* amplicons and the 3 studied isolates, we identified the following number of types for the S1–S9 spacers: 3, 5, 3, 4, 3, 2, 3, 2, and 2 types, respectively (Table). For each of spacers S1, S2, S4, and S6, a new genotype, i.e., 10, 8, 6, and 5, respectively, was identified. For each spacer, differences among genotypes mainly consisted of nucleotide substitutions. Ranges of nucleotide similarity rates among tested strains were 98.5%–100% for spacer 1, 97.4%–99.7% for spacer 2, 98.3%–99.7% for spacer 3, 99.1%–99.8% for spacer 4, 98.9%–100% for spacer 5, 97.8%–99.7% for spacer 6, 98.8%–99.6% for spacer 7, 98.3%–99.4% for spacer 8, and 97.6%–99.4% for spacer 9. Combining genotypes obtained from the 9 spacers allowed the 75 studied human *B. henselae* strains to be classified into 16 MST genotypes. Of these, 5 MST genotypes, including 64 strains, were previously known among cat isolates, i.e., types 5, 16, 22, 26, and 31. The remaining 11 MST genotypes, numbered types 40–50 including 1 human strain each, were new.

**Table:** Human-infecting *Bartonella henselae* included in this study and corresponding genotypes*

No. patients by clinical condition				Genotypes									
CSD	Endocarditis	Bacillary angiomatosis		S1	S2	S3	S4	S5	S6	S7	S8	S9	MST
21	1			5	1	1	1	2	2	2	1	1	5
1†				5	2	1	1	2	2	2	1	1	16
37				3	5	4	5	2	2	2	1	3	22
3				5	1	1	5	2	2	2	1	1	26
1				5	1	1	1	2	2	4	2	1	31
		1‡		3	5	5	4	1	5	1	1	3	40
	1§			5	6	1	1	2	2	2	1	1	41
1				5	1	1	1	5	2	4	2	1	42
1				5	8	1	1	2	2	4	2	1	43
1				5	1	1	1	2	2	4	1	1	44
1				5	5	1	5	2	2	2	1	1	45
1				5	1	4	1	2	2	2	1	1	46
1				3	1	4	1	2	2	2	1	3	47
1				3	5	4	6	2	2	2	1	3	48
1				10	1	1	1	2	2	2	1	1	49
	1			5	2	5	4	1	2	1	1	1	50

*CSD, cat-scratch disease; MST, multispacer typing. †Marseille strain. ‡Houston-1 strain. §URBHTOIE 49 strain.

Sequences from the 4 new genotypes of spacers S1, S2, S4, and S6 and the 11 new MST genotypes were deposited in the GenBank database under accession nos. EF017703 (tRNA-*Ala*/GCA-tRNA-*Ile*/AUC spacer, type 10), EF017704 (BH2865724-*dut* spacer, type 8), EF017705 (*pssA*-Oxidoreductase spacer, type 6), and EF017706 (*alr*-*gcvP* spacer, type 5). These sequences were then added to the MST Rick database.

### Phylogenetic Analysis

Phylogenetic trees obtained by using alignment of the 9 concatenated spacer sequences and the neighbor-joining, maximum parsimony, and maximum likelihood methods showed similar organizations. The 50 MST genotypes (including the 39 MST genotypes previously identified among cat isolates [*31*] and the 11 new MST genotypes identified in the present study) were grouped into 4 clusters. Two clusters were associated with previously described Houston-1 and Marseille serotypes. Cluster 1 was composed of 19 MST genotypes and contained 22 American and all 19 Asian cat isolates, and only 1 amplicon detected in the cardiac valve of the patient with endocarditis from Guadeloupe Island. Cluster 2 (Houston-1) comprised 6 MST genotypes and contained 17 European and 5 American cat isolates as well as 39 amplicons, including 38 from patients with CSD and type strain Houston-1. Cluster 3 (Marseille) included 21 MST genotypes represented by 8 European and 38 American cat isolates and 35 amplicons, including 33 from patients with CSD, 2 from patients with endocarditis. and type strain Marseille. Cluster 4 contained 13 European and 4 American cat isolates but no human amplicon.

Two genotypes ( 5, and 22,) were mainly found in the Houston-1 and Marseille clusters, respectively. Genotype 22 contained 37 human strains or amplicons and 16 European cat isolates; genotype 5 included 22 human amplicons, 7 European and 23 American cat isolates. The 3 human strains from patients with endocarditis were classified within 3 different MST genotypes, i.e., genotypes 22, 41, and 50. Type strain Houston-1, obtained from a patient with bacillary angiomatosis, exhibited the unique genotype 50, whereas type strain Marseille shared genotype 16 with an American cat isolate.

The genotypic diversity of human strains was not statistically different from that of cat isolates (16/75 vs. 39/129, p = 0.3), even when restricted to French human strains or cat isolates (14/73 vs. 6/29, p = 0.9). However, when we compared the distribution of human strains among clusters, we found that the Houston-1 and Marseille clusters contained significantly more human strains than did cluster 1 (p<0.01) and also that the Houston-1 cluster contained a significantly higher proportion of human strains than the Marseille cluster (p<0.01).

In addition to type strains Houston-1 and Marseille, the 16S rRNA genotype was known for 67 cat strains. Phylogenetic cluster 1 contained 18 type I strains; cluster Houston-1 contained 21 type I strains and 1 type II strain; and clusters Marseille and 4 contained 20 and 9 type II strains, respectively.

## Discussion

We report the successful adaptation of MST to *B. henselae* detected in human samples. Isolating *B. henselae* from CSD patients is extremely difficult (*35*). In this context, a reliable and reproducible molecular typing method, using PCR coupled to sequencing, to study the genetic diversity of *B. henselae* detected in human specimens directly, is a valuable option. Recently, MLST that used 9 housekeeping genes classified 20 cat and 17 human isolates into 7 types, with most of the 17 human isolates belonging to 1 specific genotype. That study suggested that human isolates were more homogenous than cat isolates. However, the study was limited by the small number of human isolates studied and the limited discriminatory power of MLST, which hindered in-depth exploration of the genotypic diversity of *B. henselae* (*27*). In our present study with MST, using 9 highly variable ITS exhibiting a high resolution for subtyping *B. henselae* (*31*), we investigated the genetic diversity of *B. henselae* detected in humans.

When MST was previously applied to *B. henselae* cat isolates, we found 39 distinct genotypes, 4 of which (5, 2, 22, and 35) were predominant (*31*). We identified 16 MST genotypes among the 75 *B. henselae* human strains. Of these, 59 strains (78.7%) also belonged to genotypes 5 and 22. We found no statistical difference in genotypic diversity between the 75 human strains (16 MST genotypes) and the 126 previously studied cat isolates (39 genotypes, p = 0.14) (*31*).

The addition of 11 new MST genotypes to the 39 previously identified did not modify the phylogenetic distribution of 4 main clusters (lineages) described among cat isolates (*31*). However, the human strains had a specific phylogenetic distribution. Clusters 1 and 4 contained significantly fewer human strains than did clusters Houston-1 and Marseille (p<0.01). For cluster 1, this difference may be explained by the fact that this cluster contained only cat isolates from the United States and Asia, whereas we studied mostly human strains from France. The only human strain classified within cluster 1 was detected in 1 patient from the West Indies, but we acknowledge that this single patient, although exhibiting a specific genotype, does not allow us to draw any conclusions about the distribution of *B. henselae* genotypes in this area. Therefore, estimating the MST genotypes of human strains from the West Indies, United States, and Asia and comparing them to MST genotypes classified within cluster 1 might be useful. Regarding the distribution of strains within cluster 4 (4 cat strains from the United States, 10 cat strains from France, and 3 cat strains from Germany, but no human strain), we speculate that these strains may be less pathogenic for humans or that a sampling bias occurred. Thus, additional human and cat *B. henselae* isolates or amplicons from more countries will be needed to investigate the geographic correlation between human and cat isolates.

Previously, on the basis of the polymorphisms of the *pap31* gene, among 107 *B. henselae* human strains originated from France, Zeaiter et al. identified 4 genotypes grouped into 2 lineages, Marseille, including genotypes Marseille and CAL-1, and Houston-1, including genotypes Houston-1 and ZF-1; this remains the largest genetic study of *B. henselae* human strains (*24*). Of the 107 human strains, 63 and 40 hold genotypes CAL-1 and ZF-1, respectively, which were predominant among French human strains (*24*). In our study, among 73 French human strains, 14 MST genotypes were also identified into 2 lineages, Houston-1, including 38 strains of 2 genotypes, and lineage Marseille, including 35 strains of 12 genotypes; genotypes 22 and 5 within lineages Houston-1 and Marseille, respectively, contained 37 and 22 human strains and were predominant. Thus, the phylogenetic relationships of French human strains identified by *pap31* were similar to what was found by using MST, although MST was more discriminatory than *pap31*-typing (p<0.01). However, MLST based on 9 genes later identified 7 genotypes and 3 lineages (Marseille, Houston-1, and Berlin-2) among 20 cat and 17 human isolates (*27*). In contrast, our results, which were based on a larger number and a wider distribution of both cat and human strains and the more discriminatory MST, differ from those of these and other authors in that we identified 2 lineages (lineages 1 and 4) besides Marseille and Houston-1 (*18*,*27*). Lineage 1 contained only Asian and American strains, which were not included in the study by MLST. Lineages Marseille and Houston-1 thus appear to be the main phylogenetic organization of *B. henselae* species. However, the phylogenetic organization of *B. henselae* species, as shown by both MLST and MST, was more complex than the structure of 2 main lineages (*27*,*31*). The 4 lineages based on MST provided the most detailed and reasonable illustration of the phylogenetic organization of *B. henselae* species because of its ability to show the geographic distribution of *B. henselae*. However, more strains, especially more human strains of various origin, should be studied by using MST to verify and modify this phylogenetic organization.

When we compared the MST classification to classification by 16S rRNA genotypes, we observed that 16SrRNA type I strains were restricted to clusters 1 and 2 (Houston-1), whereas most type II strains were grouped into clusters 3 (Marseille) and 4. However, a discrepancy between MST and 16S rRNA typing was observed within the Houston-1 cluster. One German cat strain classified in MST genotype 22 exhibited the 16S rRNA type II, in contrast to all other strains for which the 16S rRNA type was known, including other German strains. Such a discrepancy is consistent with the findings of Iredell et al., who demonstrated that 16S rRNA typing of *B. henselae* isolates was not entirely congruent with their lineage allocations (*27*).

In conclusion, we demonstrated, with 16 genotypes identified among 75 *B. henselae* human strains, that MST was more discriminatory than previously described methods for investigating *B. henselae* infection in humans. We did not find any statistically significant difference in genetic diversity between human and cat isolates of *B. henselae*. The studied human strains, although geographically limited, were phylogenetically organized into 2 clusters, which matched the origin of cat strains previously described as Houston-1 and Marseille clusters. Further studies incorporating strains from more diverse geographic origins and clinical features will be needed to improve our understanding of the population dynamics of *B. henselae.* We believe that MST can be a valuable tool for this purpose.

## Supplementary Material

Appendix Figure Dendrogram showing the phylogenetic organization of the 50 multispacer typing genotypes identified among 126 Bartonella henselae cat isolates and 75 B. henselae isolates detected in humans, constructed by using the neighbor-joining method. Sequences from the 9 spacers were concatenated. The scale bar represents a 1% nucleotide sequence variation. *I = 16S rDNA type I, II = 16S rDNA type II. The number in the brackets indicates the number of B. henselae strains available for determination of 16S rDNA type.

Appendix Table Primers used for amplifying and sequencing the 9 intergenic spacers
