# Supplementary figures and images for: Genetic Diversity of Bartonella henselae in Human Infection Detected with Multispacer Typing
Source: Emerg Infect Dis. 2007 Aug;13(8):1178–83. doi: 10.3201/eid1308.070085 (PMC2828084; doi:10.3201/eid1308.070085)

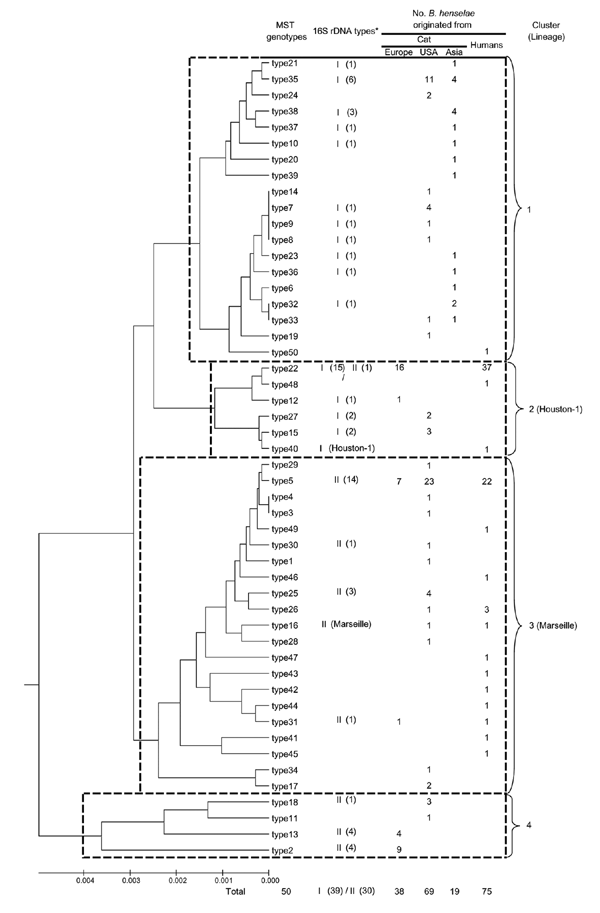

Supplement: Appendix Figure — Dendrogram showing the phylogenetic organization of the 50 multispacer typing genotypes identified among 126 Bartonella henselae cat isolates and 75 B. henselae isolates detected in humans, constructed by using the neighbor-joining method. Sequences from the 9 spacers were concatenated. The scale bar represents a 1% nucleotide sequence variation. *I = 16S rDNA type I, II = 16S rDNA type II. The number in the brackets indicates the number of B. henselae strains available for determination of 16S rDNA type. [file 07-0085_appF-s1.gif]
