# Supplementary material for: Genetic Diversity of Bartonella henselae in Human Infection Detected with Multispacer Typing
Source: Emerg Infect Dis. 2007 Aug;13(8):1178–83. doi: 10.3201/eid1308.070085 (PMC2828084; doi:10.3201/eid1308.070085)
Supplement: Appendix Table — Primers used for amplifying and sequencing the 9 intergenic spacers [file 07-0085_appT-s2.pdf]

Appendix Table. Primers used for amplifying and sequencing the 9 intergenic spacers

Spacer name	Spacer position on the genome*	Spacer size (amplicon size, bp)†	Forward primer (5´?3´)	Reverse primer (5´?3´)	Reference
tRNA-Ala/GCA-tRNA-Ile/AUC (S1)	1412349–1412683	335 (588)	TGCACAGAAAAACCTCACCTT	AGGTCGGAGGTTCAAGTCCT	This study
BH2865724-*dut* (S2)	1685859–1686289	431 (602)	GGTTTTTGCCACGGGTATTT	GGAAGTTCTAAACCTTGTCCATGG	(31)
DnaJ related protein-*cobS* (S3)	1828960–1829320	361 (490)	CAATGGAGGCAACCGTTCTT	GTGATATCGGGTACATTTTCAACTG	(31)
pssA-Oxidoreductase (S4)	609654–610228	575 (709)	GATTTTTCTTCCGTGTAGCTTTGT	TGTGCGTAAAAATCGATTCATG	(31)
carB-Cold shock protein (S5)	1292681–1293066	386 (509)	AGAAGCTATCGAAGCACTCACAAA	TGAATGAACCCGAAACCTTTAGT	(31)
alr-gcvP (S6)	1431110–1431442	333 (540)	TCAAAGAGGTGATTGGGTAGAGC	CTGTTTCACGTATTGATAATGTTGC	(31)
ftsK-Oxidoreductase (S7)	1799482–1799984	503 (594)	GCGAACCTTGAGAACTCTGCA	GGGTTTTACACCTTCATTGAGATCA	(31)
BH2864883-BH2864884 (S8)	1594026–1594377	352 (689)	GAAATTTCCTGCTGCAAAGC	GAAAATTGGGGAGGGTGTTT	This study
acpP2-Malate oxidoreductase (S9)	853898–854063	166 (296)	CAACTTCACTGATTTCTGCGATAA	CGAGGAGTGGTTAATATGACAGCT	(31)

*Intergenic spacer designations consist of the name of the 5´ open reading frame (ORF)–the name of the 3´-ORF. ORFs encoding putative proteins of unknown function have designations beginning with BH and are numbered with reference to the *Bartonella henselae* type strain, Houston-1, genome (GenBank accession no. BX897699).

†The position of the spacers on the genome and spacer sizes were deduced from the genome sequence of *B. henselae* Houston-1. These values may vary among *B. henselae* strains.
